# A Multi-Omics Approach Reveals Enrichment in Metabolites Involved in the Regulation of the Glutathione Pathway in LIN28B-Dependent Cancer Cells

**DOI:** 10.3390/ijms25031602

**Published:** 2024-01-27

**Authors:** Matteo Stocchero, Diana Corallo, Silvia Bresolin, Marcella Pantile, Paola Pirillo, Roberta Bortolozzi, Sara Menegazzo, Daniele Boso, Giampietro Viola, Eugenio Baraldi, Alessandra Biffi, Giuseppe Giordano, Sanja Aveic

**Affiliations:** 1 Department of Women and Children’s Health, University of Padova, 35128 Padova, Italy; silvia.bresolin@unipd.it (S.B.); paola.pirillo@gmail.com (P.P.); roberta.bortolozzi@unipd.it (R.B.); sara.menegazzo.3@phd.unipd.it (S.M.); giampietro.viola.1@unipd.it (G.V.); alessandra.biffi@unipd.it (A.B.); giuseppe.giordano@unipd.it (G.G.); 2 Laboratory Mass Spectrometry and Metabolomics, Istituto di Ricerca Pediatrica (IRP), Fondazione Città della Speranza, 35127 Padova, Italy; 3 Laboratory of Target Discovery and Biology of Neuroblastoma, Istituto di Ricerca Pediatrica (IRP), Fondazione Città della Speranza, 35127 Padova, Italy; m.pantile@irpcds.org (M.P.); daniele.boso@iov.veneto.it (D.B.); s.aveic@irpcds.org (S.A.); 4 Department of Pharmaceutical and Pharmacological Sciences, University of Padova, 35128 Padova, Italy; 5 Department of Dental Materials and Biomaterials Research, RWTH Aachen University Hospital, 52074 Aachen, Germany

**Keywords:** LIN28B, neuroblastoma, transcriptome, metabolome, omics integration, glutathione metabolism

## Abstract

The RNA-binding protein LIN28B, identified as an independent risk factor in high-risk neuroblastoma patients, is implicated in adverse treatment outcomes linked to metastasis and chemoresistance. Despite its clinical significance, the impact of LIN28B on neuroblastoma cell metabolism remains unexplored. This study employs a multi-omics approach, integrating transcriptome and metabolome data, to elucidate the global metabolic program associated with varying LIN28B expression levels over time. Our findings reveal that escalating LIN28B expression induces a significant metabolic rewiring in neuroblastoma cells. Specifically, LIN28B prompts a time-dependent increase in the release rate of metabolites related to the glutathione and aminoacyl-tRNA biosynthetic pathways, concomitant with a reduction in glucose uptake. These results underscore the pivotal role of LIN28B in governing neuroblastoma cell metabolism and suggest a potential disruption in the redox balance of LIN28B-bearing cells. This study offers valuable insights into the molecular mechanisms underlying LIN28B-associated adverse outcomes in neuroblastoma, paving the way for targeted therapeutic interventions.

## 1. Introduction

Neuroblastoma is the most common extracranial solid tumor and the most enigmatic malignancy in childhood. The lethality rate of neuroblastoma ranks first in pediatric oncology [1]. More than 50% of patients affected by neuroblastoma are classified as high-risk, and the prognosis for these patients is reserved due to the severe clinical, molecular, and histological parameters contributing to the limited efficacy of the currently available therapies [2]. The remaining patients belong to the very-low-, low-, or intermediate-risk groups, which have a better outcome [3]. A small percentage of neuroblastomas staged as MS, according to the International Neuroblastoma Risk Group Staging System (INRGSS) [4], eventually regress spontaneously, although the biological mechanisms behind this phenomenon are still unknown. The tumors in high-risk patients are particularly aggressive and refractory to chemotherapy. The survival rate after relapse drops dramatically below 20% [4], underscoring the imperative to identify new molecular markers that might anticipate disease progression or serve as a target for innovative and tailored (adjuvant) therapies.

At the molecular level, aggressive neuroblastoma tumor phenotypes correlate with a definite number of oncogene drivers, such as *MYCN*, *ALK*, and *LIN28B* [5]. These oncogenes have been linked with metabolic reprogramming in several solid tumors [6,7,8]. *MYCN* amplification is the most consistently observed independent genetic alteration linked to poor prognosis and treatment resistance in several pediatric malignancies, including neuroblastoma [9]. For this reason, the metabolic profile of *MYCN*-amplified neuroblastomas has been widely studied, and its reprogramming has been defined as a possible vulnerability of this cancer [10,11].

LIN28B, an RNA-binding protein, regulates numerous cellular activities during embryogenesis by regulating pluripotency and the metabolism of stem cells [12]. LIN28B plays a pivotal role in repressing the maturation and functionality of microRNAs (miRNAs) and messenger RNAs (mRNAs) in either physiological or pathological conditions. Indeed, its major function is to inhibit the processing of miRNA precursors, especially those belonging to the let-7 miRNA family, thereby preventing their accumulation during the early stages of development [13]. In addition, by inhibiting the maturation of let-7 microRNA, LIN28B suppresses several components of the PI3K-mTOR pathway associated with the regulation of insulin metabolism, which has resulted in an increased glucose uptake in transgenic mice [14]. However, its overexpression, beyond the physiologically defined timeframe, has been linked to pro-tumorigenic features in various cancers [15]. One of the mechanisms through which LIN28B may exert its role is the regulation of glycolysis. In fact, enhanced aerobic glycolysis has been observed in hepatocellular carcinomas overexpressing LIN28B, where LIN28B acts by targeting the metabolic enzyme PDH kinase 1 (PDK1) [16]. Similarly, transgenic mice overexpressing LIN28B in the liver develop hepatic cancer with higher glucose consumption compared to the surrounding normal tissue, mirroring the behavior of a subset of aggressive human hepatocellular carcinomas [17]. Increased aerobic glycolysis in cancer stem cells expressing LIN28B can also be regulated via the LIN28B/MYC/miRNA-34a-5p axis [7]. Finally, additional results revealed a novel mechanism of metabolic regulation involving the LIN28B/let-7/IGF2BP1 axis in acute myeloid leukemia [18]. In neuroblastoma, the overexpression of LIN28B correlates with stemness characteristics and increased proliferative and migratory capacities in tumor cells [19,20]. However, LIN28B’s involvement in neuroblastoma cell metabolism has not been fully elucidated, but its broader impact on tumorigenesis suggests a complex interaction with metabolic pathways, similar to its role in other cancer types.

Here, we performed integrated transcriptomics and metabolomics analyses on neuroblastoma cells with induced LIN28B (iLIN28B) protein expression to outline a LIN28B-dependent metabolic profile. Compared to the controls, iLIN28B neuroblastoma cells showed a higher rate of metabolites involved in the production of nucleotides, amino acids, and especially glutathione. Glycolytic function in iLIN28B cells was compromised in time, whereas the release of the metabolites belonging to the glutathione pathway was the most abundant late event, implying an impaired redox balance in LIN28B-overexpressing neuroblastoma cells.

## 2. Results

### 2.1. Exploratory Transcriptomics Data Analysis Highlights Potentially Relevant Transcripts for the Metabolic Diversity of iLIN28B Cells

**Figure 1:**
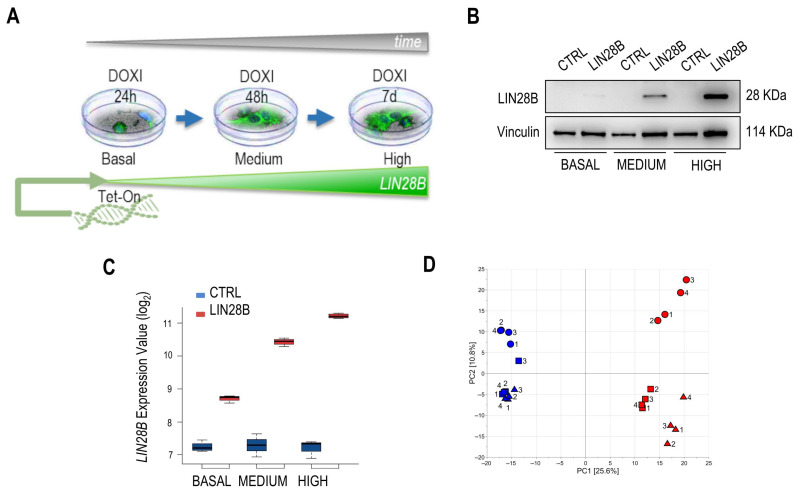
Expression analysis as a function of time in the LIN28B-inducible neuroblastoma cell model. (**A**) Schematic presentation of the doxycycline (DOXI)-inducible (Tet-On) in vitro cell model and the time-dependent regulation of the LIN28B level (basal vs. medium vs. high). (**B**) The total protein fraction has been analyzed to verify time-dependent LIN28B levels (basal, medium, and high). Vinculin is used as a loading control. kDa—molecular weight of the protein expressed in kiloDaltons. (**C**) *LIN28B* gene expression values are presented in the DOXI-induced cell system and respective CTRL cells. Y-values are shown in the log2 scale. (**D**) Score scatter plot of the PCA model obtained considering the transcriptomics data. Triangles are used to represent samples with basal iLIN28B, boxes are used for samples with medium iLIN28B, and circles are used for samples with high iLIN28B. Samples of cell lines ‘iLIN28B’ and ‘CTRL’ are colored in red and blue, respectively. The identifier of the experimental replicate is reported as a number near the symbol.

The tetracycline-controlled (Tet-On) gene expression system has been adopted for the generation of the in vitro cell model with graded *LIN28B* transcript induction (iLIN28B; Figure 1A). Immunoblot analysis and evaluation of the corresponding transcripts confirmed a time-dependent increase in iLIN28B level, defined as basal, medium, and high (Figure 1B,C). Next, we investigated the transcriptional dynamics of iLIN28B cells as a function of the induction time. A total of 21,448 gene expression variables were extracted. No outliers were detected by assuming a significance level of α = 0.05. As a result, a dataset composed of 21,448 features and 24 observations was obtained. A PCA model explaining 36.4% of the total variance was built, and the score scatter plot of the model is reported in Figure 1D. The samples belonging to CTRL cells showed negative values for the first score component PC1, whereas iLIN28B samples consistently exhibited positive values across all time points. Moreover, the time of cell culture increased along the second component PC2, suggesting that the cluster structure underlying the observations mirrored the experimental design conditions. Consequently, the global gene expression was shown to be different in iLIN28B cells at basal levels of iLIN28B, remaining different in high iLIN28B, since the factor ‘time’ influenced the transcriptome. Furthermore, the technical replication of the experiment yielded a data variation that is negligible compared to that resulting from biological effects. Univariate data analysis based on MLR revealed 4834 transcripts significantly related to LIN28B overexpression (Supplementary Figure S1A). PLS modeling showed that the design factors ‘time’ and ‘class’ were both significant. Specifically, one predictive score component was obtained to explain the factor ‘time’ (R^2^ = 0.965, *p* = 0.001), whereas the factor ‘class’ was associated with one predictive score component with MCC = 0.840 (*p* = 0.001; Supplementary Figure S1B). The observations were grouped based on the experimental condition, regardless of the experimental replicate, hence confirming the results of the exploratory data analysis. The analysis of Pearson’s correlation between the score components and measured gene expressions highlighted 3204 transcripts as relevant. Hence, merging the results of univariate and multivariate data analysis, a set of 4834 relevant transcripts, intended for subsequent joint-pathway analysis, was obtained. The validation of the in silico data was then performed using quantitative real-time PCR (qPCR) for several randomly selected genes, confirming that they were differentially expressed between those identified in CTRL neuroblastoma cells and those with a high expression of iLIN28B (Supplementary Figure S1C).

### 2.2. iLIN28B Triggers Glycolysis in Neuroblastoma Cells

**Figure 2:**
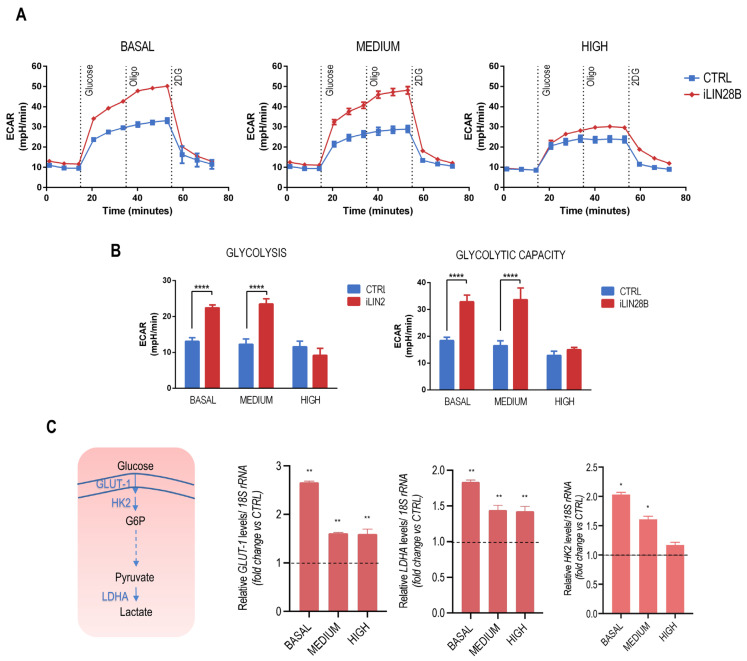
Glycolysis rate measurements in LIN28B and CTRL cells. (**A**) Measurement of the extracellular acidification rate (ECAR) was performed to assess the basal glycolysis and glycolytic capacity as a function of the iLIN28B level (in time). (**B**) Glycolysis and glycolytic capacity were derived from ECAR measurements. (**C**) A simplified scheme of the glycolytic pathway is shown on the left. Gene expression of the metabolic enzymes *GLUT-1*, *LDHA*, and *HK2* was determined in iLIN28B via qPCR and depicted as the fold change respective to CTRL neuroblastoma cells; *18S* rRNA served as an internal control. The dashed line corresponds to the relative gene expression level of the CTRL sample (RQ = 1). Data are presented as mean (±s.d.); *n* = 3. * *p* < 0.05; ** *p* < 0.01; **** *p* < 0.0001 compared to controls (Student’s *t*-test).

LIN28B is a potent regulator of host cell metabolism and aerobic glycolysis [14]. To confirm this role in our cell model, we used the Agilent Seahorse XF glycolysis stress assay, which provides a measurement of the extracellular acidification rate (ECAR) and an assessment of the glycolytic function of cells, to evaluate the impact of iLIN28B overexpression on this metabolic process. As expected, iLIN28B cells exhibited higher ECAR compared with CTRL cells by reflecting significantly higher basal glycolysis rates and glycolytic capacities (Figure 2A,B). Interestingly, this effect was dependent on the factor ‘time’ because we showed a glycolytic increase after basal growth in iLIN28B, but a decreased expression profile at a later ‘time’ corresponding to the highest level of iLIN28B. We found analogous behavior for the three critical glycolytic enzymes, *GLUT-1*, *LDHA*, and *HK2* [21], for which an immediate peak in expression was detected, followed by less-pronounced differences for cells with medium and high iLIN28B expression (Figure 2C).

### 2.3. LIN28B Defines a Metabolic Rewiring in Neuroblastoma Cells

**Figure 3:**
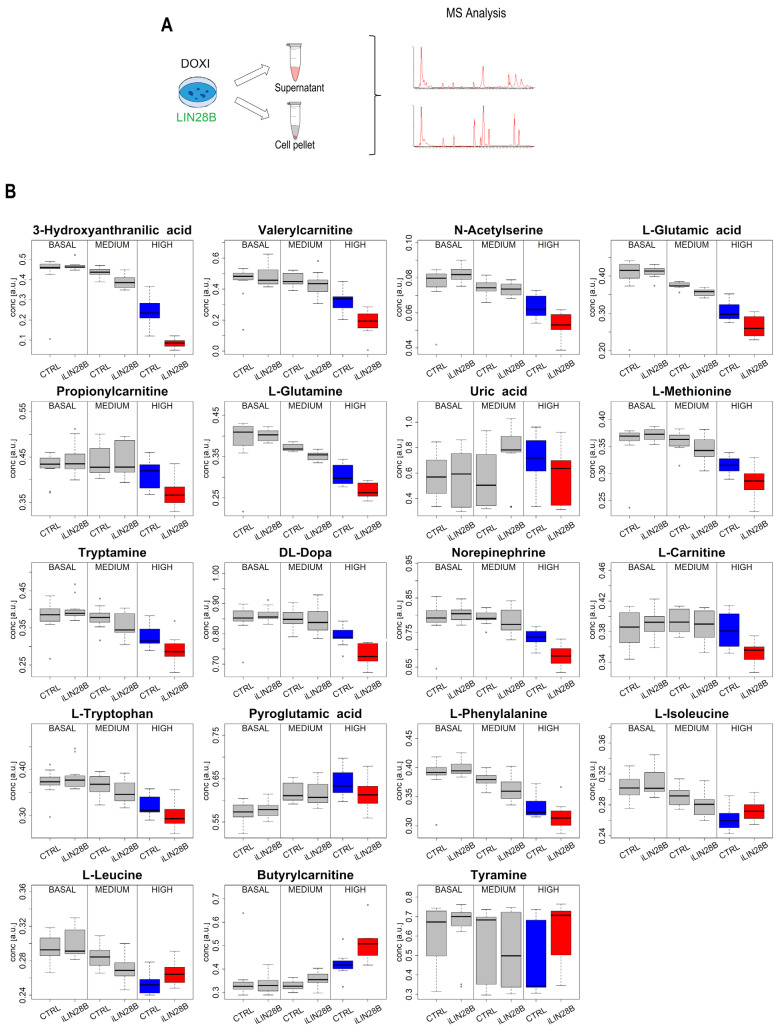
Metabolomics data. (**A**) Experimental design and sample collection for metabolomics analysis. Supernatants and cell pellets were collected from the CTRL and iLIN28B cells at basal, medium, and high iLIN28B levels. All the samples were subjected to mass spectrometry (MS) analyses. (**B**) Boxplots representing the distributions of the 19 annotated metabolites at basal, medium, and high iLIN28B levels. Significantly different levels were found in the case of high-iLIN28B samples, for which red and blue are used for ‘iLIN28B’ and ‘CTRL’, respectively.

To further dissect the global metabolic changes in iLIN28B neuroblastoma cells over ‘time’, we performed a non-targeted metabolomics analysis using ultra-high liquid chromatography coupled with high-definition mass spectrometry. Both types of samples, cell pellets, and the corresponding supernatants were analyzed. While no significant hits were discovered in the intracellular content, the metabolite profiling of the supernatant revealed potential LIN28B-associated candidates that required further investigation (Figure 3A). After data pre-processing, 1306 time mass variables were extracted. No outliers were detected, assuming a significance level of α = 0.05. As a result, a dataset composed of 1306 features and 72 observations was obtained. Exploratory data analysis based on PCA discovered a relevant effect of ‘time’ and of the ‘experimental replicate’ on the metabolic content of the supernatant, while the effect of the cell type was not highlighted (Supplementary Figure S2). MLR-based analysis and PLS modeling led to the same conclusions, proving that only the design factor ‘time’ and the blocking factor ‘experimental replicate’ were significantly associated with the metabolome.

By examining each time point independently, we discovered a significant difference between the metabolic content of iLIN28B cell supernatants compared to the CTRL counterparts in the samples with the highest iLIN28B level. Specifically, univariate data analysis based on a Mann–Whitney test controlling FDR highlighted that 74 metabolites were significantly different (Supplementary Figure S3A). An orthogonally constrained PLS for the classification model, where the blocking factor ‘experimental replicate’ was used as a constraint, with one predictive and one non-predictive score component, showed MCC = 1.000 (*p* = 0.046) and an MCC in cross-validation equal to 0.753 (*p* = 0.005) (Supplementary Figure S3B) by proving a significant effect of the cell type on the metabolome. Stability selection discovered 76 relevant metabolites. By merging the results of the univariate and multivariate data analysis, a set of 123 relevant metabolites was obtained. Among these, 19 metabolites were annotated at level 1 (Supplementary Table S1). The distributions of the annotated metabolites are represented as boxplots in Figure 3B. Within this list, 15 metabolites (L-carnitine, L-glutamic acid, L-phenylalanine, norepinephrine, pyroglutamic acid, uric acid, tryptamine, DL-dopa, L-glutamine, L-methionine, propionylcarnitine, L-tryptophan, 3-hydroxyanthranilic acid, N-acetylserine, and valerylcarnitine) were shown to be downregulated and 4 (L-isoleucine, tyramine, butyrylcarnitine, and L-leucine) were upregulated in the supernatants of high-iLIN28B samples compared to the CTRL (Supplementary Table S1).

### 2.4. Omics Data Integration Potentiates the Discovery of LIN28B-Dependent Regulatory Pathways

**Figure 4:**
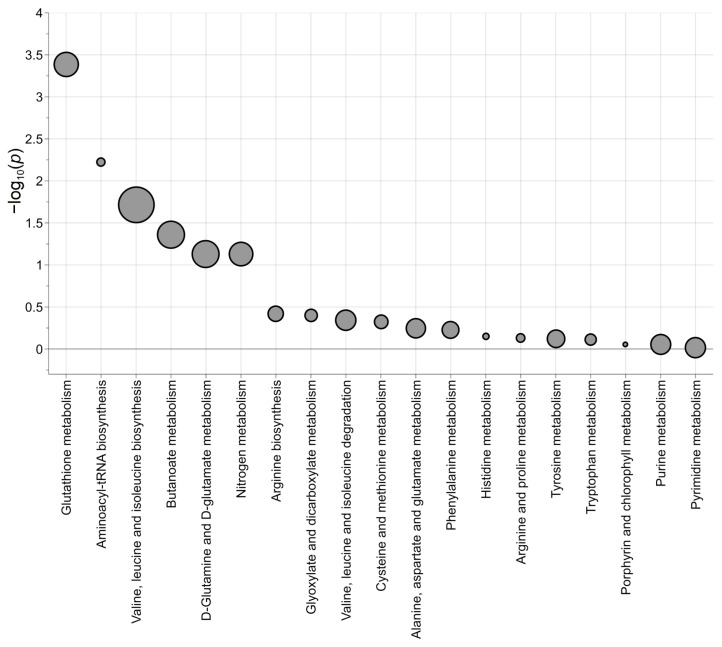
Joint pathway analysis. The negative logarithm of *p* is reported as −log_10_(*p*), while the symbol size is proportional to the statistical impact of the pathway calculated via topological analysis. Source: KEGG pathway database for *Homo sapiens*.

To seek insights into the metabolic pathways that occur in neuroblastoma cells carrying LIN28B overexpression, transcriptomics and metabolomics data have been integrated. In doing so, our aim was to obtain a deeper and more robust annotation of the metabolic pathways regulated specifically by iLIN28B. Indeed, the discovery process of the perturbed metabolic pathways under the condition of high iLIN28B expression can be improved if the information related to the gene expression is combined with that of the metabolic arrangement. Since the information arising from the transcripts can drive the analysis toward the real perturbed pathways, this may either confirm or contradict the results obtained when considering only the metabolites. We combined the 19 annotated relevant metabolites and the 4834 relevant transcripts at the metabolic pathway level by applying joint pathway analysis (Supplementary Table S2). In particular, by considering the pathways with a *p*-value inferior to 0.10, a subset of 8 metabolites (L-glutamic acid, L-glutamine, L-isoleucine, L-leucine, L-methionine, L-phenylalanine, L-tryptophan, and pyroglutamic acid) were shown to be associated with pathways related to glutathione metabolism, aminoacyl-tRNA biosynthesis, valine, leucine, and isoleucine biosynthesis, butanoate metabolism, D-glutamine, and D-glutamate metabolism, and nitrogen metabolism (Figure 4). Interestingly, glutathione metabolism was the most perturbed pathway represented (20 out of 56 features). Specifically, 2 metabolites (L-glutamic acid and pyroglutamic acid) and 18 transcripts significantly enriched this metabolic pathway (*p*-value < 0.001, adjusted-*p*-value = 0.016). All the other perturbed pathways showed adjusted *p*-values greater than 0.15, except for aminoacyl-tRNA biosynthesis with an adjusted *p*-value of 0.10. These findings indicated possible new therapeutic vulnerabilities in neuroblastoma tumor cells carrying the LIN28B oncogene. In particular, the glutathione pathway is the most appealing, since different druggable targets can be exploited for therapeutic purposes [22]. Further functional and pharmacological analyses are needed to answer the question of whether glutathione pathway inhibition may trigger therapy-related hypersensitivity in LIN28B-overexpressing neuroblastomas.

## 3. Discussion

In this study, we exploited the effects of two LIN28B-dependent score components, one explaining the factor ‘time’ and one the factor ‘class’, in regulating neuroblastoma cells’ metabolic status. In particular, we reconstructed the global metabolic pathway changes after time-dependent LIN28B overexpression by crossing two different layers of biological information: the transcriptome and the metabolome. Moreover, the experimental design adopted in this study enabled us to follow the biological effects dependent on LIN28B levels as a function of time regardless of the *MYCN* expression, which remains not significantly modulated in our system. Indeed, unlike the traditional capturing of the metabolome data at a fixed-frame snapshot, the time-based analysis has enormous potential to unveil transient metabolites, both qualitatively and quantitatively. The metabolic phenotype of cancer cells carrying the LIN28B oncogene has been the subject of intense investigation over the years, since the induced metabolic changes provide the energy and biomacromolecules necessary for tumor cell growth [23]. LIN28B is linked with cancer stemness and, due to genomic amplification, it is occasionally found overexpressed in a subset of high-risk patients with neuroblastoma. Clinically, LIN28B defines poor prognosis and adverse outcomes [19]. Through the doxycyclin-inducible cell system, we achieved different levels of LIN28B protein in vitro, thus mimicking the diversity found in human neuroblastoma specimens [19]. We confirmed the known role of LIN28B in boosting the glycolytic rate [7] as an early event in neuroblastoma cells. However, over time, the metabolic preference for this pathway appears to be replaced by other biological processes, such as glutathione metabolism. The investigation of the secreted metabolites in the cell medium highlights significant differences between high-level iLIN28B cells and the corresponding controls. In total, 19 differentially expressed metabolites were identified, 7 of them directly involved in the aminoacyl-tRNA biosynthesis pathway, which regulates protein synthesis and is involved in cancer progression [24]. Among these metabolites, 4 were upregulated and 15 were downregulated in iLIN28B supernatants. Among the upregulated metabolites, Tyramine is an essential regulator of catecholamine release from the adrenal glands. Through its cellular internalization, Tyramine stimulates the secretion of norepinephrine, a mechanism usually activated in response to stress [25]. Considering the tissue of origin of neuroblastoma, its extracellular downregulation may be associated with increased norepinephrine synthesis, a phenotype commonly used to detect and diagnose neuroblastoma in patients through an MIBG scan. L-Leucine and Isoleucine are essential amino acids produced during the pyruvic acid pathway that is involved in many biological processes, such as protein synthesis and energy production. The extracellular downregulation of Isoleucine might be the result of an increased intracellular consumption as an anaplerotic substrate [26]. Butyrylcarnitine is a product derived from the degradation of isoleucine that is found to be overexpressed in different types of human malignancies [27,28]. Its overexpression correlates to better overall survival, and its extracellular downregulation has been associated with decreased B-oxidation [29]. Our study revealed 19 metabolites significantly dysregulated in LIN28B cells that could potentially be useful in cancer diagnosis. Although the potential of cell cultures has been exploited as a model system for metabolomics studies [30], additional in vitro investigations on primary cells and samples from human malignant tissue and plasma are needed to further define the biological impact of the metabolites reported in this study. Among the 15 extracellularly downregulated metabolites, 5 (L-Phenylalanine, L-Glutamine, L-Methionine, L-Tryptophan, and L-Glutamate) are directly involved in the aminoacyl-tRNA biosynthesis pathway, the most significantly enriched pathway at this time point in iLIN28B overexpression.

Our integrative approach allowed the investigation of the overlap between impaired transcriptional and metabolic pathways in neuroblastoma cells carrying different amounts of intracellular LIN28B. By merging the identified metabolites with the transcripts that were found to be linked with iLIN28B, we identified six metabolic pathways of potential interest in neuroblastoma: glutathione metabolism, aminoacyl-tRNA biosynthesis, valine, leucine and isoleucine biosynthesis, butanoate metabolism, D-glutamine and D-glutamate metabolism, and nitrogen metabolism. The correlation between metabolome and transcriptome revealed the activation of central metabolic pathways involved in the fast proliferation of neuroblastoma cells and in the response to nutrient limitation and stress hints, such as glutathione (GSH). In particular, two metabolites and eighteen genes involved in the glutathione pathway are significantly enriched in iLIN28B cells. Maintaining proper levels of GSH is crucial, as dysregulation can lead to the manifestation of pathogenic functions. Through its role as an intracellular antioxidant, GSH protects cells from increased oxidative stress [31], and in a subset of *MYCN*-amplified neuroblastoma cells, its disruption was reported to determine cell hypersensitivity to drugs [32]. Another significantly enriched pathway obtained from both the analysis of the secreted metabolites and from the integration of transcriptomics and metabolomics data is the aminoacyl-tRNA-biosynthesis pathway. LIN28B plays an important role as a post-transcriptional regulator, influencing either the promotion or repression of translation. A recent result obtained by Tan and colleagues showed the formation of two discrete translational subpopulations based on the different levels of LIN28B protein induced in HEK293A human cells [33]. In agreement with this, it is reasonable to speculate that different levels of LIN28B might have a differential impact on protein translation in neuroblastoma cells and this could reflect the different metabolic phenotypes observed in response to a different ‘time’ of LIN28B induction.

A well-known function of LIN28B is the regulation of let-7 microRNA (miRNA) biogenesis [14,15,16]. MiRNAs play a pivotal role as master regulators of gene expression. Several studies highlight their involvement in the metabolic reprogramming of tumor cells, where various miRNAs exert both positive and negative regulation on multiple metabolic genes [34]. In particular, different miRNAs such as miR-433 and miR-214 have been documented as important regulators of the redox state in cancer [35]. We could not exclude a parallel role for direct miRNA targets of LIN28B in altered neuroblastoma metabolism. However, further studies are required to unravel the intricate relationships and pathways that may mediate the impact of LIN28B on neuroblastoma metabolism through its interaction with specific miRNAs.

Our data confirmed that the short induction of LIN28B, which led to the basal level of the protein, maintained cellular metabolism in a state of increased glycolysis and relatively low oxidative phosphorylation (OxPhos) [12]. We therefore deduced that glucose was the main carbon source for the de novo synthesis of glutamate, glutamine, and glutathione through the TCA cycle. However, a prolonged overexpression of LIN28B was not accompanied by a significant increase in glucose metabolism, suggesting that iLIN28B-level diversity enabled the formation of two distinct metabolic phenotypes in neuroblastoma cells. Overall, our data extended the metabolic role of LIN28B in regulating glucose homeostasis in neuroblastoma cells. Nevertheless, the functional and clinical significance of the observed metabolic alterations induced by LIN28B requires further experimental evaluation incorporating functional assays, analyses of patient-derived material, and in vivo studies. Establishing the clinical translatability of these findings could enhance their relevance for developing targeted therapeutic interventions in neuroblastoma patients with elevated LIN28B expression. Finally, we demonstrated that the multi-omics integration approach further facilitates the discovery of metabolic pathways in cancer cells by uncovering the dynamic temporal patterns of metabolites in human neuroblastoma cells.

## 4. Material and Methods

### 4.1. Experimental Design

Doxycycline-inducible SH-SY5Y control (CTRL) and induced LIN28B (iLIN28B) cell lines were obtained via a lentiviral infection following the protocol described previously [20]. The study design was longitudinal: both cell types were investigated by administering doxycycline for 0 h (basal LIN28B level), 48 h (medium LIN28B level), or 7 days (high LIN28B level) of in vitro culture, for a total of 6 experimental conditions. In the case of metabolomics investigation, 3 biological replicates were obtained for each condition, and the experiment was repeated 4 times, for a total of 12 independent samples in each experimental condition. For the transcriptomics investigation, 2 biological replicates for each condition and 2 experimental replicates were performed, for a total of 4 independent samples in each experimental condition.

### 4.2. Glycolysis Function Assays

The glycolytic function was measured through the Agilent Seahorse XF Glycolysis Stress Test (Agilent Technologies, Santa Clara, CA, USA. In brief, 30,000 CTRL or iLIN28B cells per well were seeded in an XF96 plate left overnight at 37 °C, in a 5% CO_2_ incubator in complete medium. The day after, medium was replaced with DMEM without glucose, L-glutamine, phenol red, sodium pyruvate, and sodium bicarbonate (Sigma-Aldrich, St. Louis, MO, USA) enriched with 2 mM of L-glutamine (pH 7.4) and after 1 h of incubation in a 37 °C non-CO_2_ incubator, the plate was transferred to the Seahorse XFe96 Extracellular Flux Analyzer to quantify the extracellular acidification rate (ECAR, in [mpH/min]) and the oxygen consumption rate (OCR, in [pmol/min]). The following final concentrations of compounds were used: glucose 10 mM (Sigma-Aldrich, St. Louis, MO, USA), Oligomycin 1 µM (Sigma-Aldrich, St. Louis, MO, USA), and 2-DG 50 mM (TCI EUROPE, Zwijndrecht, Belgium). The oxygen consumption rate (OCR) and extracellular acidification rate (ECAR) were normalized using total protein amount in each well obtained using BCA assays (Thermo Fisher Scientific, Waltham, MA, USA).

### 4.3. RNA Isolation, cDNA Synthesis, and a Real-Time Quantitative PCR (qPCR) Assay

RNA extraction was performed using TRIzol reagent (Invitrogen, Thermo Fisher Scientific, Waltham, MA, USA) and Zymo Direct-zol (Zymo Research, Freiburg im Breisgau, Germany) columns as described previously [20], and the concentration was measured using Qubit (Invitrogen, Thermo Fisher Scientific, Waltham, MA, USA). RNA quality and integrity were monitored using the RNA Nano Assay on the Agilent 2100 Bioanalyzer instrument (Agilent Technologies, Santa Clara, CA, USA). For cDNA synthesis, 1 µg of RNA was used following Superscript II (Invitrogen, Thermo Fisher Scientific, Waltham, MA, USA) protocol. Subsequently, qPCR was performed on an Applied Biosystems 7900 HT Sequence Detection System using SYBR Green PCR Master Mixture reagents (Invitrogen, Thermo Fisher Scientific, Waltham, MA, USA). Experiments were performed in triplicate. The primers used in the study were *GLUT-1* (Forward 5′-GCGGAATTCAATGCTGATGAT-3′, Reverse 5′-CAGTTTCGAGAAGCCCATGAG-3′); *LDHA* (Forward 5′-AGCCCGATTCCGTTACCT-3′, Reverse 5′-CACCAGCAACATTCATTCCA-3′); *18S* (Forward 5′-AAACGGCTACCACATCCAAG-3′, Reverse 5′-CAATTACAGGGCCTCGAAAG-3′); *NRP1* (Forward 5′-ATGCGAATGGCTGATTCAGG-3′, Reverse 5′-TCCATCGAAGACTTCCACGTAG-3′); *COL6A1* (Forward 5′-CCTCTGCCCGGACCCTCA-3′, Reverse 5′-CACGGACCCCGAGAAAACCT-3′); *HK2* (Forward 5′-CCTGGTCTCATGGACCAAGGG-3′, Reverse 5′-ACACAGCCACAATGTCGAT-3′); *GAPDH* (Forward 5′-AAGGTGAAGGTCGGAGTCAA-3′, Reverse 5′-TGAAGGGGTCATTGATGGCA-3′). Relative expression levels were determined via normalization using expression of the *18S* or *GAPDH* as endogenous control genes, and results were interpreted using the comparative ΔΔCt method. Statistical analyses were performed using GraphPad Prism 7 (GraphPad, La Jolla, CA, USA).

### 4.4. Immunoblot Assay

For total protein extraction, iLIN28B and CTRL cells were trypsinized (PAN Biotech, Aidenbach, Germany), washed with cold PBS once, and resuspended in cold lysis buffer (Biosource International, Camarillo, CA, USA) supplemented with protease and phosphatase inhibitors (Sigma-Aldrich). Immunoblotting was performed with 20 µg of the proteins quantified with Pierce BCA protein assay kit (Thermo Fisher Scientific, Waltham, MA, USA) following the manufacturer’s recommendations. Proteins were loaded on 4–20% gradient gel and SDS-PAGE (Bio-Rad, Hercules, CA, USA) was performed as described elsewhere [36]. Primary antibodies used in the study were anti-LIN28B (Cell Signaling, Danvers, CA, USA; 4196S) and anti-Vinculin (Santa Cruz, Dallas, TX, USA; sc-73264). For protein visualization, secondary horseradish-peroxidase (HRP)–conjugated antibodies (Sigma-Aldrich) were used. Enhanced chemiluminescence (ECL) Western blotting detection reagents (ECL^TM^ Select, Merck Life Science S.r.l., Milan, Italy) were used for protein band acquisition with the iBright Imaging Systems (Life Technologies, Thermo Fisher Scientific, Waltham, MA, USA).

### 4.5. Gene Expression Analysis and Data Interpretation

For gene expression analysis, 100 ng of total RNA was prepared for the transcription, hybridization, and biotin labeling according to the array protocol for the GeneChip™ Whole Transcript PLUS Reagent Kit Manual Target Preparation (Affymetrix, Thermo Fisher Scientific, Waltham, MA, USA). Samples were hybridized using the Human Clariom™ S Gene Chip Cartridge Array (Thermo Fisher Scientific, Waltham, MA, USA). CEL files were normalized using the robust multiarray averaging expression measure with Transcriptome Analysis Console (TAC Software v. 4.0.2.15, Thermo Fisher Scientific, Waltham, MA, USA). Differentially expressed genes between iLIN28B and CTRL were identified using the Significance Analysis of Microarray (SAM) algorithm coded in the samr R package (https://www.metaboanalyst.ca, accessed on 28 April 2023) [37] for any time points (i.e., 0 h, 48 h, and 7 days). The estimated percentage of false-positive predictions (i.e., false discovery rate, FDR) was obtained with 1000 permutations, and genes with an FDR < 0.01 were considered significant. The data supporting the findings of this study are available in GEO, reference number GSE252806.

### 4.6. Metabolomics Investigation

#### 4.6.1. Sample Preparation

The CTRL and LIN28B cells were grown in flasks and harvested after administering doxycycline at the indicated time points. Four pellets of about 1 × 10^6^ cells were harvested for each time point analyzed. The entire harvesting and extraction process was performed on ice. Eight ceramic beads of 1.4 mm in diameter (MagNa Lyser Green Beads, Roche, Basel, Switzerland), previously washed in MeOH, were added to each sample pellet together with 250 µL of extraction solvent (2:2:1, MeOH:EtOH:H_2_O). Samples were then lysed in the pre-cooled Homogenizer Tissue Lyser (Roche, Basel, Switzerland), through two cycles of 1 min at 25 Hz with 30 s pause between the two cycles. Then, lysates were transferred to dry ice for 15 min to favor protein precipitation. The samples were centrifuged at 1600× *g* for 3 min, and the lysis procedure was repeated two more times with 100 µL of extraction solvent at a time.

Cell supernatants were thawed on ice and mixed using a Vortex mixer for 10 s. Then, 350 µL of cold CH_3_OH were added to 50 µL of cell supernatant, left at −20 °C for 30 min, and centrifuged at 16,000× *g* for 15 min at 4 °C. Then, 100 µL of supernatant was transferred into a 200 µL well plate and evaporated to dryness under nitrogen flow, and re-dissolved in 150 µL of H_2_O:CH_3_CN (0.1% *v*/*v* HCOOH) as 80:20 ratio.

#### 4.6.2. Untargeted Metabolomics Analysis

Untargeted metabolic profiling was performed in positive and negative electrospray ionization mode on an Acquity Ultra Performance Liquid Chromatography system (Waters MS Technologies, Milford, MA, USA) coupled to a Quadrupole Time-of-Flight Synapt G2 HDMS mass spectrometer (Waters MS Technologies, Milford, MA, USA). Data were collected in IM-MSE, in a scanning range of 20–1200 *m*/*z*. Chromatography was performed using an Acquity HSS T3 (1.7 μm, 2.1 × 100 mm) column (Waters Corporation, Milford, MA, USA) kept at 50 °C. The flow rate of the mobile phase was set at 0.5 mL/min, and each sample run lasted 12 min, with 5 µL of the sample injected for each run. The gradient mobile phase consisted of H_2_O (0.1% *v*/*v* HCOOH) (phase A), and CH_3_OH: CH_3_CN (0.1 % *v*/*v* HCOOH) 90:10 (phase B). Each sample run lasted 11 min, comprising an isocratic phase of 5% B for 1 min, a linear increase to 30% B for 2.5 min, a linear increase to 95% B for 3 min, an isocratic phase of 95% B for 1.5 min, and a washout phase of 5% B for 3 min. Quality control samples (QCs) and standard solution samples (Mixes) were used to assess reproducibility and accuracy during the analysis and examine the metabolite content of the samples. The QCs were prepared from an aliquot (25 µL) of each re-dissolved sample, pooled together, and diluted to three different dilution factors (1:2, 1:3, 1:5). Blank extracts were prepared by replacing cell supernatant with 50 µL of H2O and processed as described for samples. The Mixes consisted of nine compounds of known exact mass and retention time. The QCs and Mixes were injected at regular intervals into 12 samples during the sequence, together with blank samples, to evaluate mass accuracy, retention time shift, and contamination throughout the analytical sequence. The run order of the injections was randomized to prevent any spurious classification derived from the position of the sample in the sequence.

#### 4.6.3. Data Pre-Processing

Raw data were extracted using Progenesis QI software v2.4 (Waters Corporation, Milford, MA, USA), and two datasets were generated, one for the positive ionization mode and one for the negative ionization mode. The parameters used for data extraction were optimized through the preliminary processing of the QCs. We set a filter strength of 0.025 and 0.2 to import the raw data, respectively, for positive and negative ionization mode, and a QC in the middle of the sequence as a reference for the automatic alignment of all runs in the sequence. The sensitivity of the automatic algorithm for the peak picking was set equal to 5, in a chromatographic range from 0.4 to 8.0 min. The so-called time mass variables (where time is the retention time and mass is the mass-to-charge ratio *m*/*z* of the chemical compound) were generated. Only variables with no missing data in the QCs and with intensity in the blank samples less than 1/5 of the 5th percentile of the QCs were considered. Missing data were imputed by generating a random number between zero and the minimum value recorded for that variable. Thus, linear regression models explaining the behavior of the variables in the QCs at different dilutions with the run order were calculated. The models were used to estimate local calibration curves at each run of the sequence, which were useful to calibrate the recorded variables in the samples [38]. Probabilistic quotient normalization was applied to remove dilution effects due to different sample concentrations. Finally, variables with a coefficient of variation greater than 30% in the QCs were excluded. Data obtained in positive and negative ionization modes were merged and mean-centered prior to performing data analysis.

#### 4.6.4. Variable Annotation

Variable annotation was performed by searching the Human Metabolome Database, the METLIN metabolite database, and our in-house database. The fragmentation pattern was studied by injecting, in MS/MS mode, the samples with the higher ion intensity. Annotation level was defined according to Sumner et al. [39], using 4 different levels of accuracy. Specifically, level 1 (corresponding to identified metabolite) was assigned to compounds with fragmentation patterns consistent with those of standards analyzed under identical conditions, and with MS1 signals showing differences in *m*/*z* less than 10 ppm and in retention time less than 0.2 min with respect to the standards; level 2 was attributed to metabolites with *m*/*z* less than 10 ppm with respect to compounds recorded in online databases with similar fragmentation patterns; level 3 was assigned to compounds with *m*/*z* less than 10 ppm with respect to the compounds in online databases; and level 4 was utilized for repeatable signals of the mass spectrum, with no correspondence in the databases used.

### 4.7. Statistical Analysis of Metabolomics and Transcriptomics Data

Data were investigated by applying a hierarchical strategy. As the first step, exploratory data analysis was performed. Principal component analysis (PCA) [40], which summarizes the data variation in a large dataset using a small number of score components, called principal components, built via a linear combination of the measured variables, was applied to discover cluster structures, trends, and relationships between observations and variables. Moreover, outlier detection was performed by applying the T2 test and Q-distance test based on PCA. Secondly, metabolites and transcripts were independently investigated to select the relevant features to be integrated into the final step of data analysis. The experimental design was explicitly considered. The experimental factors were the time of cell culture (factor ‘time’) and the type of cell line (factor ‘class’), whereas the responses were the gene expression (transcripts) and the metabolite concentration. The experimental replicate was considered a blocking factor (factor ‘experimental replicate’). The design matrix was calculated using the coding introduced in PLS for designed experiments [41]. Specifically, the continuous quantitative factor ‘time’ was mean-centered, whereas the factor ‘class’, whose levels were indicated as ‘CTRL’ and ‘iLIN28B’, and ‘experimental replicate’, were codified as nominal categorical factors. After coding, each block of the design matrix was scaled to unit variance. As a result, an orthogonal design matrix was obtained. Both univariate and multivariate data analysis tools were applied. Specifically, a regression model based on multivariate linear regression (MLR) was built for each measured response considering the design matrix as a matrix of the predictors. False discovery rate (FDR) was controlled by the Benjamini–Hochberg procedure [42]. Multivariate data modeling was based on projection to latent structures (PLS) regression. Specifically, the design matrix was regressed on the measured responses using suitable orthogonal constraints to guarantee that each PLS score component was related to a single factor and orthogonal to the other [41]. As a result, two score components, one explaining the factor ‘time’ and the other explaining the factor ‘class’, were calculated. A randomization test working on the residuals was implemented to assess the significance of the eigenvalue calculated at each iteration of the PLS algorithm to evaluate the significance of the score component. Only score components with significant eigenvalues were considered. Relevant features were discovered by calculating Pearson’s correlation between score components and measured responses. The significance of Pearson’s correlation coefficient was assessed via a randomization test.

In the case of single time point analysis, univariate data analysis was performed using the Mann–Whitney test, controlling FDR via the Benjamini–Hochberg procedure, whereas multivariate data analysis was based on PLS for classification with stability selection [41,42,43]. The number of score components to use was determined based on the first maximum of Matthew’s correlation coefficient (MCC) calculated via 20 repeated 5-fold cross-validations under the condition of passing the randomization test on the class response. A significance level of α = 0.05 and a control level of δ = 0.05 were assumed in PLS modeling and in FDR, respectively. In the last step of data analysis, the discovered relevant metabolites and transcripts were integrated at the pathway level by applying joint-pathway analysis. Considering the pathways of *Homo sapiens* included in the KEGG pathway database, transcripts and metabolites were directly concatenated into a single query, followed by over-representation analysis [44]. Data analysis was performed using in-house R-functions implemented via the R 4.0.4 platform (R Foundation for Statistical Computing) and joint-pathway analysis using Metaboanalyst 5.0 (www.metaboanalyst.ca accessed on 28 April 2023).

## Data Availability

The data supporting the findings of this study are available in GEO, reference number GSE252806.

## Supplementary Materials

The supporting information can be downloaded at: https://www.mdpi.com/article/10.3390/ijms25031602/s1.

## References

[1] Desai A.V., Applebaum M.A., Karrison T.G., Oppong A., Yuan C., Berg K.R., MacQuarrie K., Sokol E., Hall A.G., Pinto N. (2022). Efficacy of post-induction therapy for high-risk neuroblastoma patients with end-induction residual disease. Cancer.

[2] DuBois S.G., Macy M.E., Henderson T.O. (2022). High-Risk and Relapsed Neuroblastoma: Toward More Cures and Better Outcomes. Am. Soc. Clin. Oncol. Educ. Book.

[3] Meany H.J. (2019). Non-high-risk neuroblastoma: Classification and achievements in therapy. Children.

[4] Cohn S.L., Pearson A.D.J., London W.B., Monclair T., Ambros P.F., Brodeur G.M., Faldum A., Hero B., Iehara T., Machin D. (2009). The International Neuroblastoma Risk Group (INRG) classification system: An INRG task force report. J. Clin. Oncol..

[5] De Wilde B., Beckers A., Lindner S., Kristina A., De Preter K., Depuydt P., Mestdagh P., Sante T., Lefever S., Hertwig F. (2018). The mutational landscape of MYCN, Lin28b and ALK F1174L driven murine neuroblastoma mimics human disease. Oncotarget.

[6] Hu M., Bao R., Lin M., Han X.R., Ai Y.J., Gao Y., Guan K.L., Xiong Y., Yuan H.X. (2022). ALK fusion promotes metabolic reprogramming of cancer cells by transcriptionally upregulating PFKFB3. Oncogene.

[7] Chen C., Bai L., Cao F., Wang S., He H., Song M., Chen H., Liu Y., Guo J., Si Q. (2019). Targeting LIN28B reprograms tumor glucose metabolism and acidic microenvironment to suppress cancer stemness and metastasis. Oncogene.

[8] Tjaden B., Baum K., Marquardt V., Simon M., Trajkovic-Arsic M., Kouril T., Siebers B., Lisec J., Siveke J.T., Schulte J.H. (2020). N-Myc-induced metabolic rewiring creates novel therapeutic vulnerabilities in neuroblastoma. Sci. Rep..

[9] Ruiz-Pérez M.V., Henley A.B., Arsenian-Henriksson M. (2017). The MYCN protein in health and disease. Genes.

[10] Oliynyk G., Ruiz-Pérez M.V., Sainero-Alcolado L., Dzieran J., Zirath H., Gallart-Ayala H., Wheelock C., Johansson H.J., Nilsson R., Lehtiö J. (2019). MYCN-enhanced Oxidative and Glycolytic Metabolism Reveals Vulnerabilities for Targeting Neuroblastoma. iScience.

[11] Xia Y., Ye B., Ding J., Yu Y., Alptekin A., Thangaraju M., Prasad P.D., Ding Z.C., Park E.J., Choi J.H. (2019). Metabolic reprogramming by MYCN confers dependence on the serine-glycine-one-carbon biosynthetic pathway. Cancer Res..

[12] Zhang J., Ratanasirintrawoot S., Chandrasekaran S., Wu Z., Ficarro S.B., Yu C., Ross C.A., Cacchiarelli D., Xia Q., Seligson M. (2016). LIN28 Regulates Stem Cell Metabolism and Conversion to Primed Pluripotency. Cell Stem Cell.

[13] Gewalt T., Noh K.W., Meder L. (2023). The role of LIN28B in tumor progression and metastasis in solid tumor entities. Oncol. Res..

[14] Zhu H., Ng S.C., Segr A.V., Shinoda G., Shah S.P., Einhorn W.S., Takeuchi A., Engreitz J.M., Hagan J.P., Kharas M.G. (2011). The Lin28/let-7 axis regulates glucose metabolism. Cell.

[15] Tsialikas J., Romer-Seibert J. (2015). LIN28: Roles and regulation in development and beyond. Development.

[16] Ma X., Li C., Sun L., Huang D., Li T., He X., Wu G., Yang Z., Zhong X., Song L. (2014). Lin28/let-7 axis regulates aerobic glycolysis and cancer progression via PDK1. Nat. Commun..

[17] Nguyen L.H., Robinton D.A., Seligson M.T., Wu L., Li L., Rakheja D., Comerford S.A., Ramezani S., Sun X., Parikh M.S. (2014). Lin28b is sufficient to drive liver cancer and necessary for its maintenance in murine models. Cancer Cell.

[18] Zhou J., Bi C., Ching Y.Q., Chooi J.Y., Lu X., Quah J.Y., Toh S.H.M., Chan Z.L., Tan T.Z., Chong P.S. (2017). Inhibition of LIN28B impairs leukemia cell growth and metabolism in acute myeloid leukemia. J. Hematol. Oncol..

[19] Molenaar J.J., Domingo-Fernández R., Ebus M.E., Lindner S., Koster J., Drabek K., Mestdagh P., Van Sluis P., Valentijn L.J., Van Nes J. (2012). LIN28B induces neuroblastoma and enhances MYCN levels via let-7 suppression. Nat. Genet..

[20] Corallo D., Donadon M., Pantile M., Sidarovich V., Cocchi S., Ori M., De Sarlo M., Candiani S., Frasson C., Distel M. (2020). LIN28B increases neural crest cell migration and leads to transformation of trunk sympathoadrenal precursors. Cell Death Differ..

[21] Potter M., Newport E., Morten K.J. (2016). The Warburg effect: 80 years on. Biochem. Soc. Trans..

[22] Bansal A., Celeste Simon M. (2018). Glutathione metabolism in cancer progression and treatment resistance. J. Cell Biol..

[23] DeBerardinis R.J., Lum J.J., Hatzivassiliou G., Thompson C.B. (2008). The Biology of Cancer: Metabolic Reprogramming Fuels Cell Growth and Proliferation. Cell Metab..

[24] Zhou Z., Sun B., Nie A., Yu D., Bian M. (2020). Roles of Aminoacyl-tRNA Synthetases in Cancer. Front. Cell Dev. Biol..

[25] Mandela P., Ordway G.A. (2006). The norepinephrine transporter and its regulation. J. Neurochem..

[26] Johansen M., Bak L., Schousboe A., Iversen P., Sorensen M., Keiding S., Vilstrup H., Gjedde A., Ott P., Waagepetersen H. (2007). The metabolic role of isoleucine in detoxification of ammonia in cultured mouse neurons and astrocytes. Neurochem. Int..

[27] Halama A., Guerrouahen B.S., Pasquier J., Diboun I., Karoly E.D., Suhre K., Rafii A. (2015). Metabolic signatures differentiate ovarian from colon cancer cell lines. J. Transl. Med..

[28] Zhang J., Wu G., Zhu H., Yang F., Yang S., Vuong A.M., Li J., Zhu D., Sun Y., Tao W. (2022). Circulating Carnitine Levels and Breast Cancer: A Matched Retrospective Case-Control Study. Front. Oncol..

[29] Koves T.R., Ussher J.R., Noland R.C., Slentz D., Mosedale M., Ilkayeva O., Bain J., Stevens R., Dyck J.R.B., Newgard C.B. (2008). Mitochondrial Overload and Incomplete Fatty Acid Oxidation Contribute to Skeletal Muscle Insulin Resistance. Cell Metab..

[30] Čuperlović-Culf M., Barnett D.A., Culf A.S., Chute I. (2010). Cell culture metabolomics: Applications and future directions. Drug Discov. Today.

[31] Liu Y., Hyde A.S., Simpson M.A., Barycki J.J. (2014). Emerging Regulatory Paradigms in Glutathione Metabolism. Adv. Cancer Res..

[32] Floros K.V., Cai J., Jacob S., Kurupi R., Fairchild C.K., Shende M., Coon C.M., Powell K.M., Belvin B.R., Hu B. (2021). MYCN -amplified neuroblastoma is addicted to iron and vulnerable to inhibition of the system xc-/glutathione axis. Cancer Res..

[33] Tan F.E., Sathe S., Wheeler E.C., Nussbacher J.K., Peter S., Yeo G.W. (2019). A Transcriptome-wide Translational Program Defined by LIN28B Expression Level. Mol. Cell.

[34] Subramaniam S., Jeet V., Clements J.A., Gunter J.H., Batra J. (2019). Emergence of MicroRNAs as Key Players in Cancer Cell Metabolism. Clin. Chem..

[35] Marengo B., Pulliero A., Izzotti A., Domenicotti C. (2019). miRNA Regulation of Glutathione Homeostasis in Cancer Initiation, Progression and Therapy Resistance. MicroRNA.

[36] Aveic S., Pantile M., Seydel A., Esposito M.R., Zanon C., Li G., Tonini G.P. (2016). Combating autophagy is a strategy to increase cytotoxic effects of novel ALK inhibitor entrectinib in neuroblastoma cells. Oncotarget.

[37] Tusher V.G., Tibshirani R., Chu G. (2001). Significance analysis of microarrays applied to the ionizing radiation response. Proc. Natl. Acad. Sci. USA.

[38] Santamaria F., Montella S., Stocchero M., Pirillo P., Bozzetto S., Giordano G., Poeta M., Baraldi E. (2019). Effects of pidotimod and bifidobacteria mixture on clinical symptoms and urinary metabolomic profile of children with recurrent respiratory infections: A randomized placebo-controlled trial. Pulm. Pharmacol. Ther..

[39] Sumner L.W., Amberg A., Barrett D., Beale M.H., Beger R., Daykin C.A., Fan T.W.M., Fiehn O., Goodacre R., Griffin J.L. (2007). Proposed minimum reporting standards for chemical analysis: Chemical Analysis Working Group (CAWG) Metabolomics Standards Initiative (MSI). Metabolomics.

[40] Jolliffe I. (2002). Principal Component Analysis.

[41] Stocchero M., Locci E., D’Aloja E., Nioi M., Baraldi E., Giordano G. (2019). PLS2 in metabolomics. Metabolites.

[42] Benjamini Y., Hochberg Y. (1995). Controlling the False Discovery Rate: A Practical and Powerful Approach to Multiple Testing. J. R. Stat. Soc. Ser. B.

[43] Stocchero M. (2020). Relevant and irrelevant predictors in PLS2. J. Chemom..

[44] Pang Z., Chong J., Zhou G., De Lima Morais D.A., Chang L., Barrette M., Gauthier C., Jacques P.É., Li S., Xia J. (2021). MetaboAnalyst 5.0: Narrowing the gap between raw spectra and functional insights. Nucleic Acids Res..

