# Supplementary material for: A Multi-Omics Approach Reveals Enrichment in Metabolites Involved in the Regulation of the Glutathione Pathway in LIN28B-Dependent Cancer Cells
Source: Int J Mol Sci. 2024 Jan 27;25(3):1602. doi: 10.3390/ijms25031602 (PMC10855783; doi:10.3390/ijms25031602)
Supplement: Supplementary file 1 [file ijms-25-01602-s001.zip › Supplementary_Table_S1.pdf]

Supplementary Table S1. Identified metabolites. HMDB is the Human Metabolome DataBase identifier, ‘name’ is the name of the metabolite, p is the p-value of the Mann-Whitney test, FC[CTRL/iLIN28B] is the fold change calculated as the ratio between the median of the metabo-lite concentration in the group 'CTRL' and that of the group 'iLIN28B'.

HMDB	name	p	FC[CTRL/ iLIN28B]
HMDB0000062	L-Carnitine	1.1E-05	1.07
HMDB0000148	L-Glutamic acid	1.1E-04	1.15
HMDB0000159	L-Phenylalanine	1.9E-02	1.03
HMDB0000172	L-Isoleucine	2.5E-02	0.96
HMDB0000216	Norepinephrine	3.9E-05	1.08
HMDB0000267	Pyroglutamic acid	3.0E-04	1.03
HMDB0000289	Uric acid	3.6E-02	1.13
HMDB0000303	Tryptamine	4.7E-03	1.10
HMDB0000306	Tyramine	3.7E-02	0.49
HMDB0000609	DL-Dopa	2.6E-05	1.09
HMDB0000641	L-Glutamine	4.6E-06	1.13
HMDB0000687	L-Leucine	1.3E-02	0.95
HMDB0000696	L-Methionine	1.8E-04	1.10
HMDB0000824	Propionylcarnitine	5.2E-04	1.15
HMDB0000929	L-Tryptophan	8.3E-03	1.06
HMDB0001476	3-Hydroxyanthranilic acid	6.5E-07	2.76
HMDB0002013	Butyrylcarnitine	1.7E-03	0.82
HMDB0002931	N-Acetylserine	1.7E-04	1.17
HMDB0013128	Valerylcarnitine	2.1E-05	1.74
