# Supplementary figures and images for: A Multi-Omics Approach Reveals Enrichment in Metabolites Involved in the Regulation of the Glutathione Pathway in LIN28B-Dependent Cancer Cells
Source: Int J Mol Sci. 2024 Jan 27;25(3):1602. doi: 10.3390/ijms25031602 (PMC10855783; doi:10.3390/ijms25031602)

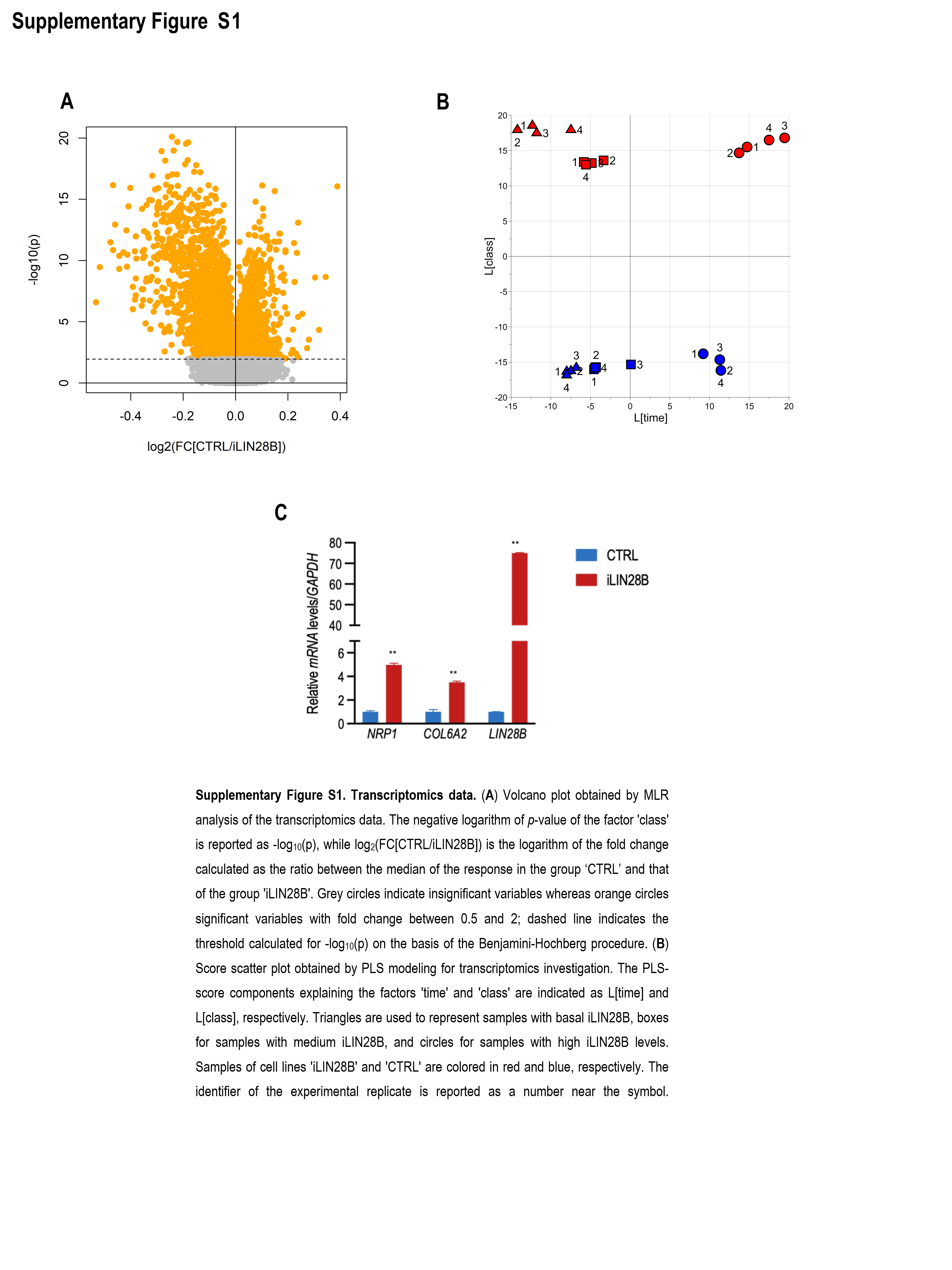

Supplement: Supplementary file 1 [file ijms-25-01602-s001.zip › Supplementary Figure S1.tif]

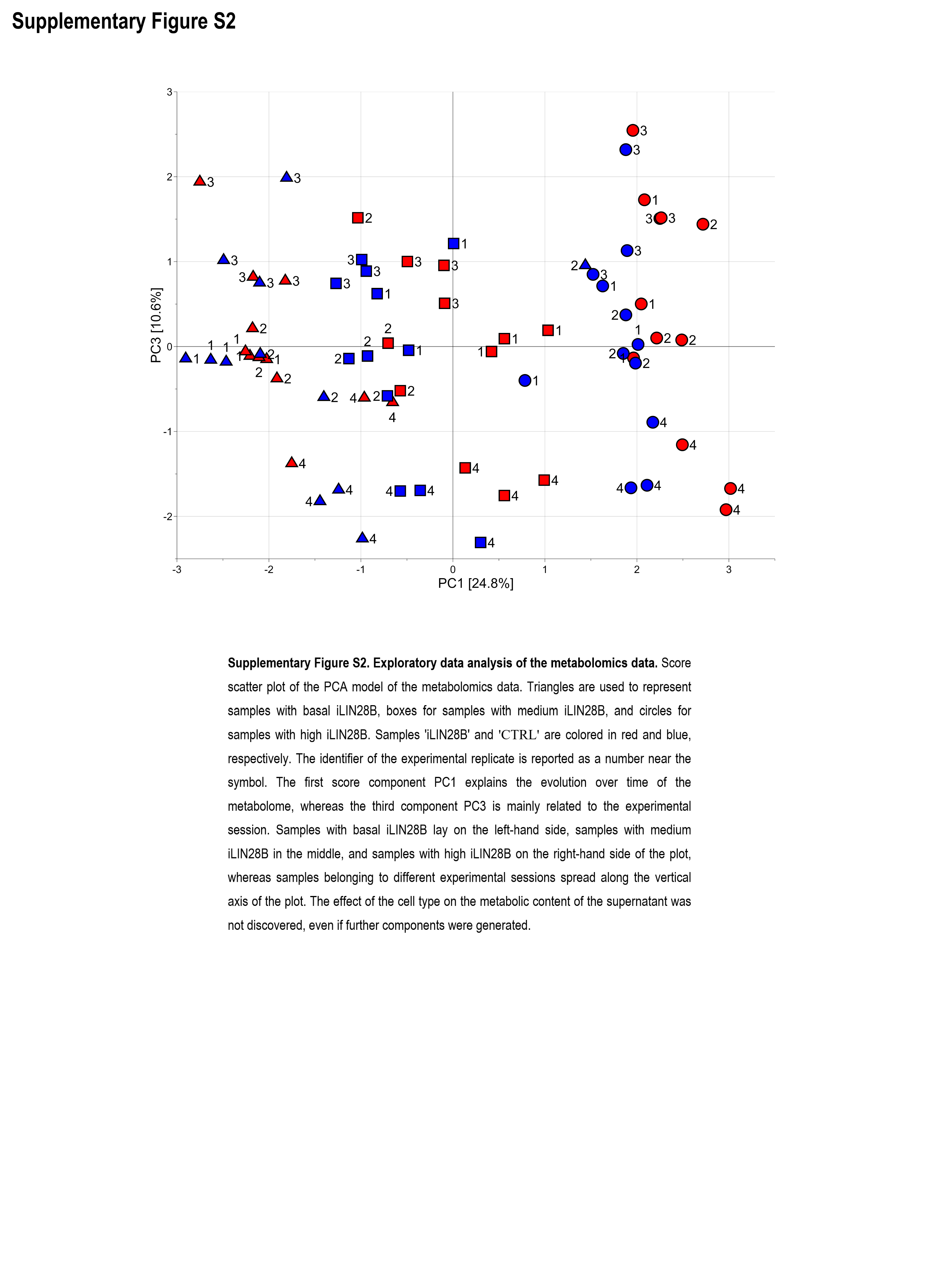

Supplement: Supplementary file 1 [file ijms-25-01602-s001.zip › Supplementary Figure S2.tif]

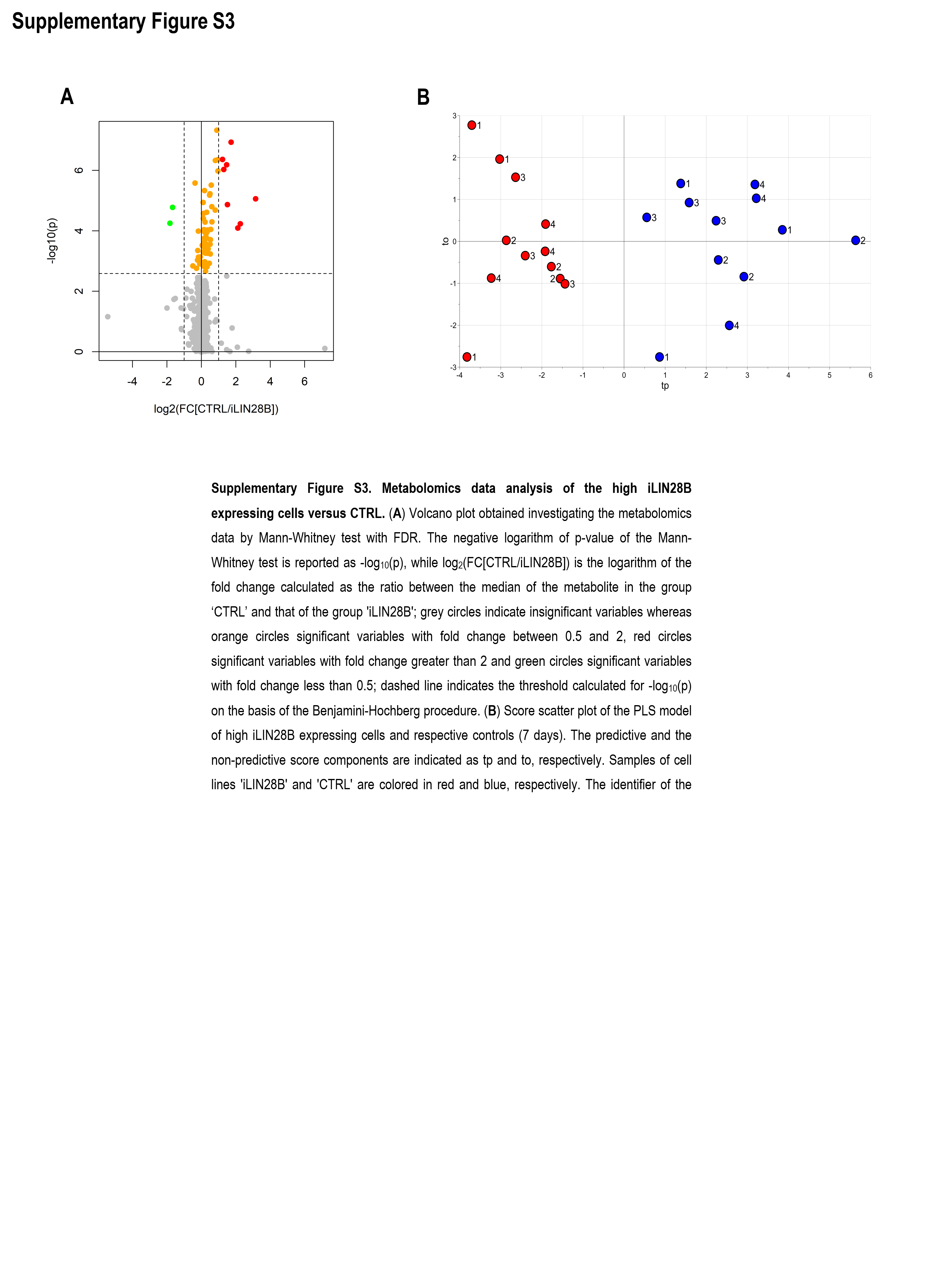

Supplement: Supplementary file 1 [file ijms-25-01602-s001.zip › Supplementary Figure S3.tif]
